# Literature review of the use of Qualitative Behaviour Assessment with a fixed list of terms

**DOI:** 10.3389/fvets.2025.1588346

**Published:** 2026-01-09

**Authors:** Irena Czycholl, Cecilie Ravn Skovlund, Björn Forkman

**Affiliations:** Department of Veterinary and Animal Sciences, University of Copenhagen, Frederiksberg, Denmark

**Keywords:** Qualitative Behaviour Assessment, emotional state, fixed list, welfare assessment, animal welfare, positive emotional state

## Abstract

Qualitative Behaviour Assessment (QBA) is a method that is used to assess emotional states in animals, either based on a list of pre-established terms (fixed list; FL) or developed through Free-Choice-Profiling. Although FL QBA was originally developed for welfare assessment of farm animals, it is nowadays also used for various species and other sectors. This is, amongst others, because QBA contributes a unique ‘whole-animal’ insight into animal experience that complements other measures and its high feasibility along with a general lack of available indicators of positive emotional state. This has led to a number of different usages and applications of FL QBA of which an overview (e.g., exact methodology used, statistical analysis, purpose and aim) so far does not exist. The aim of this review is to provide an overview of the studies that have applied FL QBA, the species it has been used for and the corresponding lists, what the QBA was used for (purpose), as well as about the various methodological approaches. Web of Science was searched between October 2023 and February 2024 for all peer-reviewed publications on FL QBA. 193 publications met inclusion criteria and were included for final review. The most common aim of use of QBA was on-farm welfare assessment, followed by measuring behavioural/emotional responses to specific events and temperament assessment/behavioural profiling. FL QBA was mainly identified for farmed animals (cattle, pigs, hens, sheep, goats, buffaloes and salmon) but also for working and companion animals (horses and donkeys, dogs and cats) as well as various exotic species in other contexts such as zoological institutions (brown bears, polar bears, elephants, dolphins, gorillas, peccaries and pampas deer). No FL QBA use was identified for laboratory animals. Methodological approaches varied greatly, including differences in term generation, observation methods (e.g., individual-vs. group observation and time spent observing), level of training and inter-observer reliability, and statistical analysis. Moreover, the level of reporting also varied greatly. In sum, this review provides a full overview of the current state of FL QBA including a list of all FL used which is important for the future development, refinement and standardisation of the method.

## 1 Introduction

Qualitative Behaviour Assessment (QBA) is currently one of the relatively few available indicators of positive welfare [see, e.g., Boissy et al. (1) and Keeling et al. (2)] and one of the few methods currently thought as directly inferring an emotional state (3). The key characteristic of QBA is that it addresses the whole dynamic animal, describing and quantifying the emotionally expressive qualities that emerge from the animal’s way of moving around its environment. Qualitative descriptors such as fearful, joyful, or energetic integrate different aspects of an animal’s demeanour and are presumed to reflect an animal’s experience of its surroundings. Thus, QBA postulates that behaviour has observable dynamic expressive qualities open to formal analysis (4).

QBA was first mentioned in literature in 2000. Based on the argument that traditional, quantitative (ethogram-based) behavioural observation methodologies may not capture information on how an animal carries out behaviour (i.e., demeanour), a qualitative approach was explored as a novel methodology for integrative animal welfare assessment (5). Qualitative approaches have been used before to identify personality traits in animal personality research, inferring on underlying constructs that are not only based on which behaviours are performed, but also on how they are performed (6). This means that instead of only quantifying certain behaviours like in traditional ethograms, QBA specifically aims to capture the quality of behaviour, i.e., the style or expressive quality. A first link between this approach and the emotional state of animals was proposed by Wemelsfelder et al. (5). In the original article, as well as in the following years to come [e.g., Wemelsfelder et al. (4, 5) and Rousing and Wemelsfelder (7)], QBA was based on Free-Choice-Profiling (FCP) methodology, in which multiple observers freely generate terms to describe animals’ behavioural expressivity, usually based on video clips. In short, in Free Choice Profiling, observers use their own words to describe the expressive quality of the behaviours they see. A group of observers observes animals (usually from video clips) and then each observer writes down descriptive terms that in his or her opinion describe best the expressive quality of behaviours observed (e.g., descriptors like curious, relaxed, fearful). Then the same observers, using their self-generated descriptor list, rate the expressivity of observed animals on a Visual Analogue Scale (VAS) ranging from ‘minimum’ (expression absent) to ‘maximum’ (expression strongly dominant). Because everyone uses different words, the data is analysed using a statistical method called Generalised Procrustes Analysis (GPA). This technique finds common patterns in the ratings, despite the differences in vocabulary (4, 5, 8).

In a widely cited literature review on measuring positive emotions in animals (1), QBA is mentioned as one potential methodology to measure positive emotional state of animals and for potential inclusion in welfare assessment protocols, although the authors also highlight the general problem of validating such indicators of positive affect. Moreover, as studies suggested good reliability [e.g., (4, 9–11)] along with the fact that not many (feasible) indicators for the positive emotional state had been described [e.g., (1)], QBA was included as a measure of positive emotion within the Welfare Quality project (WQ) (development of feasible on-farm welfare assessment protocols). In order to enhance the feasibility of the QBA method, here, for the first time, the development of QBA fixed lists (FL), as ready-to-use lists of terms, is described (9–11). In the FL approach, the list of terms is pre-established based on existing research (sometimes further developed and refined in further studies), or on consultation with suitable species experts and stakeholders [e.g., (12)], and is not, as with FCP, chosen freely by the observer(s) who end up using the list. This standardisation means that a FL QBA can be carried out by a single observer.

After inclusion in the WQ, QBA has also been extended to other welfare assessment frameworks and protocols, especially as measure for the criterion ‘positive emotional state’ [e.g., (13–16)]. Because FL QBA is now a part of various welfare assessment schemes [e.g., WQ, Animal Welfare Indicators (AWIN), Shelter Quality (SQP)] and thus the use of FL QBA is sometimes not obvious in these studies, the exact number of studies that have used FL QBA is unclear. The methods used to develop the QBA lists vary, as does the context in which it has been used. Although some literature reviews on QBA already exist, these have so far focused on its potential use in welfare assessment protocols (17–21) and were focused on a specific group of animal species and/or on the usefulness of QBA as a tool for specific contexts [e.g., inclusion in Australian livestock industry (18) or for zoo animal welfare assessment (19)]. Moreover, these were not systematic reviews. The aim of the present review is to provide a structured overview of the application of QBA in studies using the FL approach, covering all species that a FL QBA has been developed for, as well as the uses (aims) of the method. The focus on FL QBA was chosen because this approach is most relevant for welfare assessment tools (i.e., on-farm/on-site use) due to its higher feasibility compared to FCP. Therein, we did not limit on specific purposes of use of FL QBA, but aimed to provide an overview of use of FL QBA in all areas of current research. The specific research questions we aim to answer are: (1) On which animal species has the FL QBA been carried out so far? (2) How were the FLs developed? (3) What was the aim of studies using the FL QBA? (4) How was the FL QBA applied? (5) How were QBA results analysed statistically? Providing such an overview is useful for guiding further developments of the method; the review’s focus will be on identifying methodological concerns for further discussion and research. However, as this is not a comprehensive review of QBA research, it will not address whether the listed QBA FL studies have used QBA successfully or not.

## 2 Materials and methods

### 2.1 Search methods

The electronic database Web of Science (WoS) was searched for relevant publications on QBA. This was carried out between October 2023 and February 2024. After initial scoping to detect the best possible search word combination, different searches were carried out for specific species, or groups of animals, to ensure covering the most common species within farmed, companion, experimental and wild animals. Specifically, this included cattle, buffaloes, pigs, poultry, sheep, goats, horses, dogs, cats, fish, experimental animals, as well as wild and exotic animals. For each species or animal group, two searches were applied. The first search was specified by the keywords ‘qualitative + behav* + assessment’ and/or *‘QBA’*, supplemented by (i.e., also including) relevant species-specific terms (for example, species-specific terms for horse consisted of ‘*horse* OR equ* OR pon* OR *foal* OR *filly* OR *mare* OR *stallion* OR *gelding*’). The keywords of the second search included ‘*welfare + assessment’* and/or ‘Welfare + Quality*’ and/or *‘AWIN’*, along with the species-specific terms of the first search. The second search was added because QBA commonly is part of existing welfare assessment schemes such as WQ or AWIN and related publications, which in some cases, were missed by the first search. All search strings were specified to search in ‘*topic’* (includes title, abstract and author keywords) with no limitation on publication year. For experimental and wild animals, one broad search was made for each category owing to the large number of species belonging to these categories (for experimental animals, specific searches for rats, mice, hamsters, rabbits, guinea pigs were also included). Finally, the species-specific searches were supplemented by a broad search without species specific terms with the search string ‘qualitative + behav* + assessment OR *QBA* OR *welfare + assessment* OR Welfare + Quality* OR *AWIN’* to ensure all relevant publications were identified.

### 2.2 Inclusion and exclusion criteria

Title and abstract of all publications appearing in the searches were initially screened against inclusion and exclusion criteria (if the information could not be obtained from the title or abstract, the full text was screened). Publications that met all the following inclusion criteria were included in the review: (i) applied QBA as part of the study’s methodology (either focused entirely on QBA or included it as part of a larger objective), (ii) used the FL approach (either exclusively or as a second step to FCP for, e.g., term list development), (iii) published in a peer-reviewed journal, (iv) available in English, and (v) available in full. Any duplicate publications (i.e., publications that were already included) were excluded. Consequently, only original research publications utilising QBA based on the FL approach as defined by Wemelsfelder et al. (9), Wemelsfelder (10), Wemelsfelder et al. (11) (based on the respective authors’ claim and interpretation) were included. In addition publications that reported using an existing welfare assessment protocol of which QBA is an established part of (e.g., WQ, AWIN, SQP) were also included in the review, even if QBA was not specifically mentioned in the text.

### 2.3 Extraction of information

Selected parameters related to the studies’ methodology and results were extracted from the included publications. These parameters included information on the aim of the QBA, the animals used (e.g., species assessed, number of individuals, and life stages), information on the assessors (e.g., experience with species, QBA training received), the QBA method [e.g., term list development, time spent observing the animals, length of the visual analogue scale (VAS)], statistical methods (e.g., whether data suitability criteria were met, whether principal component analysis (PCA) was carried out and number of extracted principal components). Database searches, initial review of publications against inclusion and exclusion criteria, and extraction of parameters on publication-level were carried out approximately evenly distributed by the three authors. Fourteen randomly selected papers were reviewed independently beforehand to assure sufficient agreement in extraction (100%) between the three authors.

## 3 Results

The searches resulted in 193 included articles which ranged from the years 2011–2023. The last search without species-specific terms did not result in any additional articles. The studies and key results are presented in two tables: Table 1 presents the species, the setting, the life stage and the aim for which the QBA was used. Table 2 presents the experimental procedure of the same studies, i.e., origin of the QBA list of terms used, observer training, observation method and time, length of the VAS and whether QBA scores are analysed at PC or term level. Supplementary Table 1 contains the terms used in the studies on cattle, pigs and poultry, Supplementary Table 2 contains terms for sheep and goats, Supplementary Table 3 contains the same for horses and donkeys, and finally Supplementary Table 4 contains the same for dogs. The majority of the studies were done on production animals. More than half (54.4%) of the studies were on either cattle (34.7%, mainly dairy cattle) or pigs (19.7%). 11.9% of the studies were done on poultry and another 10.9% on small ruminants. On equids, encompassing working, farmed and companion animals alike, 8.8% of the studies were carried out. 8.2% of the studies were carried out on dogs (7.2%) and cats (1.0%). The remaining studies (5.2%) were done on zoo and aquaria animals or fish. No studies were found on experimental animals.

**Table 1:** Overview of the identified studies utilising fixed-list (FL) Qualitative Behaviour Assessment (QBA), the species the method was applied on, the setting and life stage of the animals during the study, and the aim of the QBA comprised into four general categories.

Reference	Species	Setting	Life stage	Aim of QBA^1^
Adamie et al. (81)	Cattle	Farm (dairy production)	Adult	General welfare assessment/emotional state (WQ)
Andreasen et al. (23)	Cattle	Farm (dairy production)	Adult	General welfare assessment/emotional state (WQ)
Andreasen et al. (82)	Cattle	Farm (dairy production)	Adult	General welfare assessment/emotional state (WQ)
Andric et al. (83)	Cattle	Farm (dairy production)	Adult	General welfare assessment/emotional state (WQ)
Armbrecht et al. (84)	Cattle	Farm (dairy production)	Adult	General welfare assessment/emotional state (WQ)
Barry et al. (85)	Cattle	Farm (dairy production)	Adult	General welfare assessment/emotional state (WQ)
Bokkers et al. (43)	Cattle	Farm (dairy production)	Adult	General welfare assessment/emotional state (WQ)
Brscic et al. (86)	Cattle	Farm (dairy production)	Juvenile	General welfare assessment/emotional state (WQ)
Bugueiro et al. (87)	Cattle	Farm (dairy production)	Adult	General welfare assessment/emotional state (WQ)
Bugueiro et al. (88)	Cattle	Farm (dairy production)	Adult	General welfare assessment/emotional state (WQ)
Ceballos et al. (51)	Cattle	Farm (dairy production)	Adult	Emotional/behavioural response or change to event
Chen et al. (89)	Cattle	Farm (experimental setting, meat production)	Juvenile	Temperament/behavioural profile
Coignard et al. (90)	Cattle	Farm (dairy production)	Adult	General welfare assessment/emotional state (WQ)
Coignard et al. (91)	Cattle	Farm (dairy production)	Adult	General welfare assessment/emotional state (WQ)
Collins et al. (92)	Cattle	Farm (dairy production)	Adult	General welfare assessment/emotional state (WQ)
Collins et al. (93)	Cattle	Farm (dairy production)	Adult	General welfare assessment/emotional state (WQ)
Cooke et al. (65)	Cattle	Farm (meat production)	Adult	General welfare assessment/emotional state (WQ)
Cooke et al. (94)	Cattle	Farm (meat production)	Juvenile and adult	General welfare assessment/emotional state (WQ)
de Andrade Kogima et al. (95)	Cattle	Farm (dairy production)	Adult	General welfare assessment/emotional state (WQ)
des Roches (25)	Cattle	Farm (experimental setting, dairy production)	Adult	General welfare assessment/emotional state (WQ)/ emotional/behavioural response or change to event
de Graaf et al. (96)	Cattle	Farm (dairy production)	Adult	General welfare assessment/emotional state (WQ)
de Rosa et al. (97)	Cattle	Farm (dairy production)	Adult	General welfare assessment/emotional state (WQ)
de Vries et al. (98)	Cattle	Farm (dairy production)	Adult	General welfare assessment/emotional state (WQ)
de Vries et al. (99)	Cattle	Farm (dairy production)	Adult	General welfare assessment/emotional state (WQ)
de Vries et al. (100)	Cattle	Farm (dairy production)	Adult	General welfare assessment/emotional state (WQ)
des Roches et al. (101)	Cattle	Farm (dairy production)	Adult	General welfare assessment/emotional state (WQ)
Dos Santos et al. (102)	Cattle	Farm (dairy production)	Adult	General welfare assessment/emotional state (WQ)
Ebinghaus et al. (37)	Cattle	Farm (dairy production)	Adult	Emotional/behavioural response or change to event
Ebinghaus et al. (50)	Cattle	Farm (dairy production)	Adult	General welfare assessment/emotional state
Ebinghaus et al. (38)	Cattle	Farm (dairy production)	Adult	Emotional/behavioural response or change to event
Ebinghaus et al. (103)	Cattle	Farm (dairy production)	Adult	General welfare assessment/emotional state
Ebinghaus et al. (104)	Cattle	Farm (dairy production)	Adult	General welfare assessment/emotional state (WQ)
Ellingsen et al. (105)	Cattle	Farm (dairy production)	Juvenile	General welfare assessment/emotional state (WQ)
Garro-Aguilar et al. (106)	Cattle	Farm (dairy production)	Adult	General welfare assessment/emotional state (WQ)
Gieseke et al. (107)	Cattle	Farm (dairy production)	Adult	General welfare assessment/emotional state (WQ)
Gois et al. (22)	Cattle	Farm (meat production)	Juvenile	Temperament/behavioural profile
Grimard et al. (108)	Cattle	Farm (dairy production)	Adult	General welfare assessment/emotional state (WQ)
Gutmann et al. (109)	Cattle	Farm (dairy production)	Adult	General welfare assessment/emotional state (WQ)
Hernandez et al. (110)	Cattle	Farm (dual purpose)	Adult	General welfare assessment/emotional state (WQ)
Hernandez et al. (111)	Cattle	Farm (dual purpose)	Adult	General welfare assessment/emotional state (WQ)
Hulsmann et al. (112)	Cattle	Farm (meat production)	Juvenile	Temperament/behavioural profile
Kaurivi et al. (27)	Cattle	Farm (meat production)	Adult	General welfare assessment/emotional state (WQ)
Kirchner et al. (113)	Cattle	Farm (meat production)	Adult	General welfare assessment/emotional state (WQ)
Kirchner et al. (114)	Cattle	Farm (meat production)	Adult	General welfare assessment/emotional state (WQ)
Krug et al. (115)	Cattle	Farm (dairy production)	Adult	General welfare assessment/emotional state (WQ)
Lutz et al. (116)	Cattle	Farm (dairy production)	Adult	General welfare assessment/emotional state (WQ)
Molina et al. (117)	Cattle	Farm (dairy production)	Adult	General welfare assessment/emotional state (WQ)
Popescu et al. (118)	Cattle	Farm (dairy production)	Adult	General welfare assessment/emotional state (WQ)
Popescu et al. (119)	Cattle	Farm (dairy production)	Adult	General welfare assessment/emotional state (WQ)
Rizzuto et al. (46)	Cattle	Rodeo	Juvenile	Emotional/behavioural response or change to event
Russell et al. (120)	Cattle	Farm (experimental, dairy production)	Adult	Emotional/behavioural response or change to event
Sant’Anna and da Costa (121)	Cattle	Farn (meat production)	Juvenile	Temperament/behavioural profile
Schmitz et al. (122)	Cattle	Farm (dairy production)	Adult	Emotional/behavioural response or change to event
Schulz et al. (123)	Cattle	Farm (dairy production)	Adult	General welfare assessment/emotional state (WQ)
Thomann et al. (124)^*^	Cattle	Farm (dairy production)	Not reported	General welfare assessment/emotional state (WQ)
Tremetsberger et al. (125)	Cattle	Farm (dairy production)	Adult	General welfare assessment/emotional state (WQ)
Tremetsberger et al. (126)	Cattle	Farm (dairy production)	Adult	General welfare assessment/emotional state (WQ)
Valente and Stilwell (127)	Cattle	Farm (various)	Not reported	General welfare assessment/emotional state (WQ)
van Eerdenburg et al. (128)	Cattle	Farm (dairy production)	Adult	General welfare assessment/emotional state (WQ)
Vucemilo et al. (129)	Cattle	Farm (dairy) production	Adult	General welfare assessment/emotional state (WQ)
Wagner et al. (130)	Cattle	Farm (dairy) production	Adult	General welfare assessment/emotional state (WQ)
Wagner et al. (131)	Cattle	Farm (dairy production)	Adult	General welfare assessment/emotional state (WQ)
Zhitia et al. (132)	Cattle	Farm (dairy) production	Adult	General welfare assessment/emotional state (WQ)
Zuliani et al. (133)	Cattle	Farm (dairy production)	Adult	General welfare assessment/emotional state (WQ)
da Silva et al. (26)	Cattle (Zebu)	Farm (dairy production)	Juvenile	General welfare assessment/emotional state
de Rosa et al. (134)	Buffalo	Farm (dairy production)	Adult	General welfare assessment/emotional state (WQ)
Napolitano et al. (34)	Buffalo	Farm (dairy production)	Adult	Other (c-QBA: evaluation of changes in animal behaviour during an observation/response to an event)
Serrapica et al. (36)	Buffalo	Farm	Adult	Other (c-QBA: evaluation of changes in animal behaviour during an observation/response to an event)
Brandt et al. (135)	Pig	Farm (meat production)	Juvenile	General welfare assessment/emotional state (WQ)
Camerlink et al. (136)	Pig	Experimental setting	Juvenile	General welfare assessment/emotional state (WQ)
Cardona et al. (137)	Pig	Farm (experimental, meat production)	Juvenile	General welfare assessment/emotional state/ Emotional/behavioural response/change to event
Cardona et al. (138)	Pig	Farm (meat production)	Juvenile	General welfare assessment/emotional state/ Emotional/behavioural response/change to event
Carreras et al. (139)	Pig	Farm (meat production)	Juvenile	General welfare assessment/emotional state (WQ)
Carroll et al. (52)	Pig	Farm (experimental, meat production)	Juvenile	General welfare assessment/emotional state
Clarke et al. (140)	Pig	Farm (meat production)	Adult	General welfare assessment/emotional state (WQ)
Czycholl et al. (141)	Pig	Farm (meat production)	Juvenile	General welfare assessment/emotional state (WQ)
Czycholl et al. (142)	Pig	Farm (meat production)	Juvenile	General welfare assessment/emotional state (WQ)
Czycholl et al. (62)	Pig	Farm (meat production)	Juvenile	General welfare assessment/emotional state (WQ)
Czycholl et al. (143)	Pig	Farm (meat production)	Juvenile	General welfare assessment/emotional state (WQ)
Czycholl et al. (144)	Pig	Farm (meat production)	Juvenile	General welfare assessment/emotional state (WQ)
Duijvesteijn et al. (145)	Pig	Farm (meat production)	(Juvenile)	General welfare assessment/emotional state (WQ)
Friedrich et al. (146)	Pig	Farm (meat production)	Adult	General welfare assessment/emotional state (WQ)
Friedrich et al. (147)	Pig	Farm (meat production)	Juvenile and adult	General welfare assessment/emotional state (WQ)
Friedrich et al. (148)	Pig	Farm (meat production)	Juvenile and adult	General welfare assessment/emotional state (WQ)
Friedrich et al. (149)	Pig	Farm (meat production)	Juvenile and adult	General welfare assessment/emotional state (WQ)
Friedrich et al. (150)	Pig	Farm (meat production)	Adult	General welfare assessment/emotional state (WQ)
Hubbard and Scott (151)	Pig	Farm (meat production)	Adult	General welfare assessment/emotional state (WQ)
Kang et al. (152)	Pig	Farm (meat production)	Juvenile	General welfare assessment/emotional state (WQ)
Losada-Espinosa et al. (153)	Pig	Farm (meat production)	All	General welfare assessment/emotional state (WQ)
Martin et al. (154)	Pig	Farm (meat production)	Juvenile	General welfare assessment/emotional state (WQ)
Martinez et al. (155)	Pig	Farm (meat production)	Juvenile	General welfare assessment/emotional state (WQ)
Meyer-Hamme et al. (156)	Pig	Farm (meat production)	Juvenile	General welfare assessment/emotional state (WQ)
Munsterhjelm et al. (157)	Pig	Farm (meat production)	Juvenile and adult	General welfare assessment/emotional state (WQ)
Munsterhjelm et al. (158)	Pig	Farm (meat production)	Juvenile and adult	General welfare assessment/emotional state (WQ)
Oldham et al. (47)	Pig	Farm (experimental, meat production)	Juvenile	Emotional/behavioural response/change to event
Rocha et al. (159)	Pig	Farm (meat production)	Juvenile	General welfare assessment/emotional state (WQ)
Schmitt et al. (160)	Pig	Farm (meat production)	Juvenile	General welfare assessment/emotional state (WQ)
Schmitt et al. (161)	Pig	Farm (meat production)	Juvenile	General welfare assessment/emotional state (WQ)
Temple et al. (162)	Pig	Farm (meat production)	Juvenile	General welfare assessment/emotional state (WQ)
Temple et al. (163)	Pig	Farm (meat production)	Juvenile	General welfare assessment/emotional state (WQ)
Temple et al. (164)	Pig	Farm (meat production)	Juvenile	General welfare assessment/emotional state (WQ)
Termatzidou et al. (165)	Pig	Farm (meat production)	Juvenile	General welfare assessment/emotional state (WQ)
Thomann et al. (124)^*^	Pig	Farm (meat production)	Juvenile and adult	General welfare assessment/emotional state (WQ)
Vitali et al. (166)	Pig	Farm (meat production)	Juvenile	General welfare assessment/emotional state (WQ)
Vitali et al. (167)	Pig	Farm (meat production)	Juvenile	General welfare assessment/emotional state (WQ)
Wiseman-Orr et al. (168)	Pig	Farm (meat production)	Juvenile	General welfare assessment/emotional state (WQ)
Bassler et al. (169)	Chickens	Farm (meat production)	Juvenile	General welfare assessment/emotional state (WQ)
Buijs et al. (170)	Chickens	Farm (meat production)	Juvenile	General welfare assessment/emotional state (WQ)
Chen et al. (171)	Chickens	Farm (meat production)	Juvenile	General welfare assessment/emotional state (WQ)
de Jong et al. (172)	Chickens	Farm/slaughter plant (meat production)	Juvenile	General welfare assessment/emotional state (WQ)
di Marcantonio et al. (173)	Chickens	Farm (meat production)	Juvenile	General welfare assessment/emotional state (WQ)
Federici et al. (174)	Chickens	Farm (meat production)	Juvenile	General welfare assessment/emotional state (WQ)
Granquist et al. (175)	Chickens	Farm (meat production)	Juvenile	General welfare assessment/emotional state (WQ)
He et al. (176)	Chickens	Farm (egg production)	Adult	General welfare assessment/emotional state (WQ)
Iannetti et al. (177)	Chickens	Farm (meat production)	Juvenile	General welfare assessment/emotional state (WQ)
Iannetti et al. (178)	Chickens	Farm (meat production)	Juvenile	General welfare assessment/emotional state (WQ)
Li et al. (179)	Chickens	Farm (meat production)	Juvenile	General welfare assessment/emotional state (WQ)
Muri et al. (180)	Chickens	Farm (meat production)	Juvenile	General welfare assessment/emotional state (WQ)
Nenadovic et al. (181)	Chickens	Farm (egg production)	Adult	General welfare assessment/emotional state (WQ)
Plitman et al. (182)	Chickens	Farm (meat production)	Juvenile	General welfare assessment/emotional state (WQ)
Sans et al. (183)	Chickens	Farm (meat production)	Juvenile	General welfare assessment/emotional state (WQ)
Sans et al. (24)	Chickens	Farm (meat production)	Juvenile	General welfare assessment/emotional state (WQ)
Sans et al. (184)	Chickens	Farm (meat production)	Juvenile	General welfare assessment/emotional state (WQ)
Sans et al. (185)	Chickens	Farm (meat production)	Juvenile	General welfare assessment/emotional state (WQ)
Souza et al. (186)	Chickens	Farm (meat production)	Juvenile	General welfare assessment/emotional state (WQ)
Souza et al. (12)	Chickens	Farm (experimental and commercial, meat production)	Juvenile	General welfare assessment/emotional state
Tuyttens et al. (187)	Chickens	Farm (meat production)	Juvenile	General welfare assessment/emotional state (WQ)
Vasdal et al. (188)	Chickens	Farm (meat production)	Juvenile	General welfare assessment/emotional state (WQ)
Vasdal et al. (189)	Chickens	Farm (egg production)	Adult	General welfare assessment/emotional and/or behavioural change/response
Bodas et al. (190)	Sheep	Farm (meat production)	Juvenile	General welfare assessment/emotional state (AWIN)
Collins et al. (191)	Sheep	Farm/pre-export (wool production)	Adult	General welfare assessment/emotional state
Diaz-Lundahl et al. (192)	Sheep	Farm (various)	Adult	General welfare assessment/emotional state
Hernandez et al. (193)	Sheep	Farm (grazing, wool production)	Not reported	General welfare assessment/emotional state
Mialon et al. (30)	Sheep	Farm (experimental, meat production)	Juvenile	General welfare assessment/emotional state (AWIN)
Muri and Stubsjøen (194)	Sheep	Farm	Adult	General welfare assessment/emotional state
Phythian et al. (195)	Sheep	Farm (various settings)	All	General welfare assessment/emotional state
Phythian et al. (196)	Sheep	Farm	Juvenile and adult	General welfare assessment/emotional state
Stubsjøen et al. (197)	Sheep	Farm	Adult	General welfare assessment/emotional state (FåreBygg project)
Willis et al. (29)	Sheep	Sea transport (wool production)	All	General welfare assessment/emotional state
Battini et al. (198)	Goat	Farm (dairy production)	Adult	General welfare assessment/emotional state (AWIN)
Battini et al. (199)	Goat	Farm (dairy production)	Adult	General welfare assessment/emotional state (AWIN)
Battini et al. (200)	Goat	Farm (dairy production)	Adult	General welfare assessment/emotional state
Battini et al. (57)	Goat	Farm (dairy production)	Adult	General welfare assessment/emotional state (AWIN)
Can et al. (201)	Goat	Farm (dairy production)	Adult	General welfare assessment/emotional state (AWIN)
Can et al. (202)	Goat	Farm (dairy production)	Adult	General welfare assessment/emotional state (AWIN)
Costa et al. (203)	Goat	Farm (experimental, feedlot system)	(Adult)	General welfare assessment/emotional state
Grosso et al. (204)	Goat	Farm (dairy production)	Adult	General welfare assessment/emotional state
Muri et al. (205)	Goat	Farm (dairy production)	Adult	General welfare assessment/emotional state
Muri et al. (206)	Goat	Farm (dairy production)	Adult	General welfare assessment/emotional state
Napolitano et al. (35)	Goat	(Farm)	Juvenile	Other (c-QBA: evaluation of changes in animal behaviour during an observation/response to an event)
Czycholl et al. (207)	Horse	Farm/boarding stable	Adult and senior	General welfare assessment/emotional state (AWIN)
Czycholl et al. (208)	Horse	Farm (various stables)	Adult and senior	General welfare assessment/emotional state (AWIN)
Czycholl et al. (209)	Horse	Farm/boarding stable (various)	Adult	General welfare assessment/emotional state (AWIN)
Dai et al. (210)	Horse	Farm/boarding stable	Adult	Emotional/behavioural response to an event
Gronqvist et al. (68)	Horse	Not reported	Adult	Other (description of expressive behaviour; valence and arousal)
Harvey et al. (53)	Horse	Wild	All	General welfare assessment/emotional state
Jaramillo et al. (28)	Horse	Racing	Juvenile and adult	Emotional/behavioural response to an event
Minero et al. (211)	Horse	Farm (various facilities)	Adult	General welfare assessment/emotional state
Mullan et al. (212)	Horse	Public grazing land	All	General welfare assessment/emotional state
Popescu et al. (213)	Horse	Farm (privately owned stallions)	Adult	General welfare assessment/emotional state (AWIN)
Rowland et al. (214)	Horse	Traveller and gypsy owned horses	Adult	General welfare assessment/emotional state
Ruet et al. (215)	Horse	Riding school	Adult	Emotional/behavioural response to an event (AWIN)
Ruet et al. (216)	Horse	Riding school	Adult	Emotional/behavioural response to an event (AWIN)
Dai et al. (217)	Donkey	Farm	Adult	General welfare assessment/emotional state (AWIN)
Dai et al. (218)	Donkey	Farm (dairy production)	Adult and senior	General welfare assessment/emotional state (AWIN)
Gonzalez et al. (219)	Donkey	Not reported	Juvenile and adult	Emotional/behavioural response to an event
Minero et al. (220)	Donkey	Farm (various)	Juvenile and adult	General welfare assessment/emotional state
Arena et al. (54)	Dog	Shelter	Adult	General welfare assessment/emotional state
Arena et al. (55)	Dog	Shelter	Adult	General welfare assessment/emotional state (SQP)
Barnard et al. (221)	Dog	Shelter	Adult	General welfare assessment/emotional state
Berteselli et al. (222)	Dog	Shelter	All	General welfare assessment/emotional state (SQP)
Berteselli et al. (223)	Dog	Shelter	Not applicable	General welfare assessment/emotional state (SQP)
Cuglovici and Amaral (224)	Dog	Shelter	Adult	General welfare assessment/emotional state (SQP)
Harvey et al. (225)	Dog	Shelter	Adult and senior	Emotional/behavioural response to an event
Menchetti et al. (31)	Dog	Shelter	Not reported	Emotional/behavioural response or change to event
Pedersen and Malm (48)	Dog	Pedagogical school dogs	Adult	Emotional/behavioural response or change to event
Raudies et al. (226)	Dog	Shelter	Adult	General welfare assessment/emotional state
Shaw et al. (227)	Dog	Privately owned companion dogs (test facility)	Adult and senior	Emotional and/or behavioural change/response
Stubsjøen et al. (228)	Dog	Shelter	Not reported	General welfare assessment/emotional state (SQP)
Stubsjøen et al. (229)	Dog	Shelter	Not reported	General welfare assessment/emotional state (SQP)
Heritier et al. (230)	Dog	Shelter	Not applicable	General welfare assessment/emotional state (SQP)
Travnik and Sant’Anna (231)	Cat	Shelter	Not reported	Temperament/behavioural profile
Travnik et al. (232)	Cat	Shelter	Not reported	Temperament/behavioural profile
Jarvis et al. (233)	Atlantic salmon	Farm (hatchery and rearing unit)	Juvenile	General welfare assessment/emotional state
Wiese et al. (44)	Atlantic salmon	Experimental setting	Juvenile	Emotional/behavioural response or change to event
Stagni et al. (234)	Brown bear	Sanctuary	Various	General welfare assessment/emotional state
Delfour et al. (32)	Dolphin	Not reported	Adult and juvenile	Other (qualitative behavioural scoring; emotional/behavioural response or change to event)
Yon et al. (235)	Elephant (Asian and African)	Zoo	Various	General welfare assessment/emotional state
Dobrikj et al. (236)	Elephant (Asian and African)	Zoo	Not reported	General welfare assessment/emotional state
Gartland et al. (33)	Gorilla	Zoo	Adult	Other (qualitative behavioural scoring)
Munerato et al. (45)	Pampas deer	Wild	Adult	Emotional/behavioural response or change to event
Skovlund et al. (237)	Polar bear	Zoo	Various	General welfare assessment/emotional state
Nogueira et al. (238)	White lipped pecari and collared pecari	Farm (commercial hunting)	Adult	Temperament/behavioural profile

WQ, Welfare Quality; AWIN, European Animal Welfare Indicators; SQP, Shelter Quality Protocol. ^1^The use of existing QBA lists from established welfare protocols are indicated by brackets of the respective protocol.

* Same study, including cattle and pig.

**Table 2:** Information on the experimental procedure of the identified studies, including the origin of the used QBA list of terms, level of observer training, observation method and time, number of animals assessed, length of the VAS and the analysis level of QBA outcomes.

Reference	Origin QBA list of terms^1^	Observer training^2^	Live or video-based observation	No. of individuals per assessment^3^	Time per assessment^4^	Length of VAS (mm)	Analysis level
Adamie et al. (81)	WQ	No training reported	Live	Not reported	20 min	125	WQ aggregation (PC level)
Andreasen et al. (23)	WQ; own creation	Official training (WQ); no training	Live	Not reported	20 min	125	PC level
Andreasen et al. (82)	WQ; own creation	Official training (WQ); no training	Live	Not reported	20 min	125	WQ aggregation (PC level)
Andric et al. (83)	WQ	No training reported	Live	Not reported (according to WQ)	Not reported (approx. 2.5–10 min)	125	WQ aggregation (PC level)
Armbrecht et al. (84)	WQ	Official training (WQ)	Live	Not reported	20 min	125	WQ aggregation (PC level)
Barry et al. (85)	WQ; own creation	Official training (WQ)	Live	Whole group	Not reported	125	WQ aggregation (PC level)
Bokkers et al. (43)	WQ	Other training; no training	Video	Not reported	2 min	125	Term and PC level
Brscic et al. (86)	WQ	Other training	Live	Not reported	20 min	125	PC level
Bugueiro et al. (87)	WQ	Official training (WQ)	Live	Not reported	20 min	125	WQ aggregation (PC level)
Bugueiro et al. (88)	WQ	Official training (WQ)	Live	Not reported	20 min	125	WQ aggregation (PC level)
Ceballos et al. (51)	Gois et al. (22) and Sant’Anna and da Costa (121)	Other training	Video	1	Not reported	125	PC level
Chen et al. (89)	Sant’Anna and da Costa (121)	No training reported	Live	1	0.5 min	136	Term level
Coignard et al. (90)	WQ	Official training (WQ)	Live	Not reported	20 min	125	WQ aggregation (PC level)
Coignard et al. (91)	WQ	Other training	Live	Not reported	20 min	125	WQ aggregation (PC level)
Collins et al. (92)	WQ	No training reported	Live	Not reported	2.5–10 min	125	Not reported
Collins et al. (93)	WQ	Official training (WQ)	Live	Not reported (according to WQ)	2.5–10 min	125	PC level
Cooke et al. (65)	WQ	Other training	Live; Video	Not reported	10 min	125	Term and PC level
Cooke et al. (94)	WQ	Other training	Live	Whole group	10 min	125	PC level
de Andrade Kogima et al. (95)	WQ	No training reported	Live	Not reported	20 min	125	Term level
des Roches (25)	WQ; own creation	Other training	Live	Individual	5 min	125	PC level
de Graaf et al. (96)	WQ	Official training (WQ)	Live	Not reported	20 min	125	WQ aggregation (PC level)
de Rosa et al. (97)	WQ	Official training (WQ)	Live	Not reported	20 min	125	WQ aggregation (PC level)
de Vries et al. (98)	WQ	Official training (WQ)	Live	Not reported (according to WQ)	20 min	125	WQ aggregation Term level
de Vries et al. (99)	WQ	Official training (WQ)	Live	Whole group	20 min	125	WQ aggregation (PC level)
de Vries et al. (100)	WQ	Official training (WQ)	Live	Not reported	20 min	125	WQ aggregation (PC level)
des Roches et al. (101)	WQ; own creation	Official training (WQ)	Live	Not reported	20 min	125	WQ aggregation (PC level)
Dos Santos et al. (102)	WQ	Other training	Live	Not reported	60 min	125	PC level
Ebinghaus et al. (37)	Focus group; own creation	Other training	Live	1	Not reported	125	PC level
Ebinghaus et al. (50)	Ebinghaus et al. (37)	Other training	Live	1	Not reported	125	PC level
Ebinghaus et al. (38)	Ebinghaus et al. (37)	Other training	Live	1	Not reported	125 (QBA App)	PC level
Ebinghaus et al. (103)	Ebinghaus et al. (37)	Other training	Live	Not reported	Not reported	125	PC level
Ebinghaus et al. (104)	WQ	No training reported	Live	Not reported	Not reported	Not reported	PC level
Ellingsen et al. (105)	WQ	No training reported	Live	1	10–20 min	125	PC level
Garro-Aguilar et al. (106)	WQ	Official training (WQ)	Live	Not reported (according to WQ)	2.5–10 min	125	WQ aggregation (PC level)
Gieseke et al. (107)	WQ	Official training (WQ)	Live	Not reported	20 min	125	WQ aggregation (PC level)
Gois et al. (22)	Sant’Anna and da Costa (121); own creation	Other training	Live	1	5 s	Not reported	Term and PC level
Grimard et al. (108)	WQ	Official training (WQ)	Live	Not reported	20 min	125	WQ aggregation (PC level)
Gutmann et al. (109)	WQ	Other training	Video	Not reported	4 min	125	Term and PC level
Hernandez et al. (110)	WQ	No training reported	Live	Whole group	20 min	125	WQ aggregation (PC level)
Hernandez et al. (111)	WQ	Official training (WQ)	Live	Not reported	20 min	125	WQ aggregation (PC level)
Hulsmann et al. (112)	Sant’Anna and da Costa (121); own creation	No training reported	Live	1	Not reported	136	Term level
Kaurivi et al. (27)	WQ; own creation	No training reported	Live	Whole group	20 min	125	Not reported
Kirchner et al. (113)	WQ	Official training (WQ)	Live	Whole group	20 min	125	WQ aggregation (PC level)
Kirchner et al. (114)	WQ	Official training (WQ)	Live	Whole group	20 min	125	WQ aggregation (PC level)
Krug et al. (115)	WQ	No training reported	Live	Whole group	20 min	125	WQ aggregation (PC level)
Lutz et al. (116)	WQ	Official training (WQ)	Live	Not reported (according to WQ)	3.5–10 min	125	Not reported (According to WQ)
Molina et al. (117)	WQ	No training reported	Live	Not reported	20 min	125	WQ aggregation (PC level)
Popescu et al. (118)	WQ	No training reported	Live	Whole group	20 min	125	WQ aggregation (PC level)
Popescu et al. (119)	WQ	Other training	Live	Not reported	20 min	125	WQ aggregation (PC level)
Rizzuto et al. (46)	WQ; own creation	Other training	Video	1	Not reported	Survey software	Term and PC level
Russell et al. (120)	WQ	Other training	Live	48	20 min	125	PC level
Sant’Anna and da Costa (121)	WQ; own creation	No training reported	Live	1	30 s	Not reported	PC level
Schmitz et al. (122)	Ebinghaus et al. (50)	Other training	Live	1	Not reported	125	PC level
Schulz et al. (123)	WQ	Official training (WQ)	Live	Whole group	20 min	125	WQ aggregation (PC level)
Thomann et al. (124)*	WQ	No training reported	Live	Not reported (according to WQ)	Not reported (according to WQ)	Not reported (according to WQ)	PC level
Tremetsberger et al. (125)	WQ	No training reported	Live	Not reported	20 min	125	WQ aggregation (PC level)
Tremetsberger et al. (126)	WQ	No training reported	Live	Not reported	20 min	125	WQ aggregation (PC level)
Valente and Stilwell (127)	WQ	No training reported	Live	Not reported	20 min	125	WQ aggregation (PC level)
van Eerdenburg et al. (128)	WQ	Official training (WQ)	Live	Not reported	20 min	125	WQ aggregation (PC level)
Vucemilo et al. (129)	WQ	No training reported	Live	17–20	20 min	Not reported	Term level
Wagner et al. (130)	WQ	Official training (WQ)	Live; Video	Not reported	20 min	125	WQ aggregation (PC level)
Wagner et al. (131)	WQ	Official training (WQ)	Live	Not reported	20 min	125	WQ aggregation (PC level)
Zhitia et al. (132)	WQ	Official training (WQ)	Live	Not reported	20 min	125	WQ aggregation (PC level)
Zuliani et al. (133)	WQ	Official training (WQ)	Live	Whole group	20 min	125	WQ aggregation (PC level)
da Silva et al. (26)	WQ; literature; own creation	No training reported	Video	1	3 min	125	PC level
De Rosa et al. (134)	WQ (for cattle)	Official training (WQ)	Live	25–64	2.5–20 min	125	Term and PC level
Napolitano et al. (34)	Napolitano et al. (35); own creation	Other training	Video	1	150 s	100	(Term level) (c-QBA)
Serrapica et al. (36)	Napolitano et al. (35); own creation	Other training	Video	1	2 min	100	(Term level) (c-QBA)
Brandt et al. (135)	WQ	Not reported	Live	Sample of herd	3.5–10 min	125	WQ aggregation (PC level)
Camerlink et al. (136)	WQ; Duijvesteijn et al. (145); own creation	Other training	Video	1	1 min	125	PC level
Cardona et al. (137)	WQ	Other training	Video	5	3–5 min	125	Term and PC level
Cardona et al. (138)	WQ	Other training	Video	10–12	1–5 min	125	Term and PC level
Carreras et al. (139)	WQ	Other training	Live	11	10 min	125	WQ aggregation (PC level)
Carroll et al. (52)	Not reported	Not reported	Live; Video	1	Not reported	100	PC level
Clarke et al. (140)	WQ	Other training	Video	15–18	1 min	100	Term and PC level
Czycholl et al. (141)	WQ	Official training (WQ)	Live	80–240	3.5–5 min	125	Term level
Czycholl et al. (142)	WQ	Official training (WQ)	Live	100–200	3.5–5 min	125	Term level and WQ aggregation (PC level)
Czycholl et al. (62)	WQ	Official training (WQ)	Live; Video	Not reported	3.5–20 min	125	Term and PC level
Czycholl et al. (143)	WQ	Official training (WQ)	Live	Sample of herd	3.5–10 min	125	WQ aggregation (PC level)
Czycholl et al. (144)	WQ	Official training (WQ)	Live	Sample of herd	3.5–5 min	125	WQ aggregation (PC level)
Duijvesteijn et al. (145)	WQ; own creation	No training reported	Video	1	1–2 min	125	PC level
Friedrich et al. (146)	WQ	Official training (WQ)	Live	Sample of herd	3.5–5 min	125	Term and PC level
Friedrich et al. (147)	WQ	Official training (WQ)	Live	Sample of herd	2.5–10 min	125	Term and PC level
Friedrich et al. (148)	WQ	Official training (WQ)	Live	Not reported	Not reported	Not reported	Not reported
Friedrich et al. (149)	WQ	Official training (WQ)	Live	Sample of herd	2.5–10 min	125	WQ aggregation (PC level)
Friedrich et al. (150)	WQ	Official training (WQ)	Live	Sample of herd	2.5–10 min	125	Term and PC level
Hubbard and Scott (151)	WQ	No training reported	Live	Not reported	2.5–10 min	125	Term
Kang et al. (152)	WQ	No training reported	Live	Sample of herd	2.5–10 min	125	Term level; WQ aggregation (PC level)
Losada-Espinosa et al. (153)	WQ	No training reported	Live	Sample of herd	2.5–10 min; 3.5–10 min	125	WQ aggregation (PC level)
Martin et al. (154)	WQ	Official training (WQ)	Live	Sample of herd	2.5–10 min	125	WQ aggregation (PC level)
Martinez et al. (155)	WQ	Official training (WQ)	Live	Sample of herd	2.5–10 min	125	WQ aggregation (PC level)
Meyer-Hamme et al. (156)	WQ	Official training (WQ)	Live	100–200	50	125	WQ aggregation (PC level)
Munsterhjelm et al. (157)	WQ	Official training (WQ)	Live	Sample of herd	2.5–10 min	125	PC level
Munsterhjelm et al. (158)	WQ	Other training	Live	Sample of herd	2.5–10 min	125	PC level
Oldham et al. (47)	WQ; Duijvesteijn et al. (145) and Rutherford et al. (3)	Other training	Video	2	30 s	125	PC level
Rocha et al. (159)	WQ	Other training	Live	Sample of herd	2.5	125	WQ aggregation (PC level)
Schmitt et al. (160)	WQ	No training reported	Live	12	20	125	Term and PC level; WQ aggregation (PC level)
Schmitt et al. (161)	WQ	No training reported	Live	Not reported	2 min	125	PC level
Temple et al. (162)	WQ	Other training	Live	Sample of herd	2.5 min	125	Term level
Temple et al. (163)	WQ	Other training	Live	Sample of herd	2.5 min	125	PC level
Temple et al. (164)	WQ	No training reported	Live	100–200	2.5 min	125	PC level
Termatzidou et al. (165)	WQ	No training reported	Live	10	2	/	(Term level)
Thomann et al. (124)*	Not reported	Not reported	Not reported	Not reported	Not reported	Not reported (according to WQ)	Not reported
Vitali et al. (166)	WQ	Official training (WQ)	Live	Not reported	5	125	WQ aggregation (PC level)
Vitali et al. (167)	WQ	Other training	Live	Not reported	5	125	Term and PC level
Wiseman-Orr et al. (168)	/	No training reported	/	/	/	100; absence/presence	Not reported
Bassler et al. (169)	WQ	Official training (WQ)	Live	Whole group	20	125	WQ aggregation (PC level)
Buijs et al. (170)	WQ	Other training	Live	Whole group	Not reported	Not reported	WQ aggregation (PC level)
Chen et al. (171)	WQ	Not reported	Live	Whole group	Not reported	Not reported	WQ aggregation (PC level)
de Jong et al. (172)	WQ	Other training	Live	Whole group	Not reported	Not reported	WQ aggregation (PC level)
di Marcantonio et al. (173)	WQ	No training reported	Live	Whole group	Not reported	Not reported	WQ aggregation (PC level)
Federici et al. (174)	WQ	Other training	Live	Whole group	Not reported	Not reported	WQ aggregation (PC level)
Granquist et al. (175)	WQ	No training reported	Live	Whole group	Not reported	Not reported	WQ aggregation (PC level)
He et al. (176)	WQ	Other training	Live	Whole group	20 min	Not reported	WQ aggregation (PC level)
Iannetti et al. (177)	WQ	No training reported	Live	Whole group	Not reported	Not reported	WQ aggregation (PC level)
Iannetti et al. (178)	WQ	No training reported	Live	Whole group	Not reported	Not reported	WQ aggregation (PC level)
Li et al. (179)	WQ	No training reported	Live	Whole group	Not reported	Not reported	WQ aggregation (PC level)
Muri et al. (180)	WQ	Other training	Live	Whole group	Not reported	Not reported	PC level
Nenadovic et al. (181)	WQ	No training reported	Live	Whole group	Not reported	Not reported	Term level
Plitman et al. (182)	WQ	No training reported	Live	Whole group	Not reported	Not reported	WQ aggregation (PC level)
Sans et al. (183)	WQ; own creation	No training reported	Live	100	5 min	125	Term level
Sans et al. (24)	WQ; Souza et al. (12)	Official training (WQ)	Live	Whole group	10 min	125	PC level
Sans et al. (184)	WQ; Souza et al. (12)	Official training (WQ)	Live	Whole group	10 min	125	PC level
Sans et al. (185)	WQ; own creation	No training reported	Live	Whole group	10 min	125	PC level
Souza et al. (186)	WQ	Other training	Live	Whole group	10	125	WQ aggregation (PC level)
Souza et al. (12)	Focus group; own creation	Other training	Video	Group	1 min	125	Term and PC level
Tuyttens et al. (187)	WQ	Official training (WQ)	Live	Whole group	20 min	125	WQ aggregation (PC level)
Vasdal et al. (188)	WQ	Official training (WQ)	Live	Whole group	Not reported	Not reported	WQ aggregation (PC level)
Vasdal et al. (189)	WQ; own creation	Official training (WQ)/Other training	Live	Whole group	20 min	125	PC level
Bodas et al. (190)	AWIN	No training reported	Live	Not reported	Not reported (according to AWIN)	Not reported according to Grosso et al. (204)	PC level
Collins et al. (191)	Literature; own creation	Other training	Video	(20)	30–45 s	100	PC level
Diaz-Lundahl et al. (192)	Literature Muri and Stubsjøen (194); farm observations; focus group; own creation	Other training	Video	111	2 min	125	PC level
Hernandez et al. (193)	AWIN; focus group; own creation	Other training	Live	9–2,000	20 min	Not reported	PC level
Mialon et al. (30)	AWIN	Other training	Video	(7; pen)	45 s	125	PC level
Muri and Stubsjøen (194)	Literature; focus group; own creation	Other training	Live; Video	3–26; 43–109	2 min; 20 min	125	Term and PC level
Phythian et al. (195)	Previous study (FCP); focus group; own creation	Official training	Video	1—whole group	1 min	125	PC level
Phythian et al. (196)	Phythian et al. (195)	Other training	Live	77	30	125	PC level
Stubsjøen et al. (197)	Literature Muri and Stubsjøen (194); own creation	Other training	Live	Whole group	20 min	Not reported	PC level
Willis et al. (29)	Literature; own creation	Not reported	Live	Whole group	5–8 min	100	(PC level)
Battini et al. (200)	Grosso et al. (204)	Other training	Live	72.28 ± 7.40	10–20	125	PC level
Battini et al. (198)	AWIN	Other training	Live	Not reported	Not reported	Not reported	Not reported
Battini et al. (199)	AWIN	Official training (AWIN)	Live	7–192	Not reported	Not reported	PC level
Battini et al. (57)	AWIN	Official training (AWIN)	Live	Whole group	10	Not reported (according to AWIN)	PC level
Can et al. (201)	AWIN	Other training	Live	Whole group	10–20	Not reported	Term and PC level
Can et al. (202)	AWIN	Other training	Live	12.5–167	Not reported (according to AWIN)	Not reported (according to AWIN)	/
Costa et al. (203)	Grosso et al. (204)	Not reported	Live	8	Not reported	Not reported	Term level
Grosso et al. (204)	Literature; focus group; own creation	Other training	Live	Whole herd	10–20	125	Term and PC level
Muri et al. (205)	WQ (dairy cows); own creation	No training reported	Live	11–173	20	Not reported	Term level
Muri et al. (206)	Not reported; own creation	Other training	Live	Whole group	Not reported	Not reported	Term level
Napolitano et al. (35)	Focus group; own creation	Other training	Video	9–10	90 s	100	(Term level) (c-QBA)
Czycholl et al. (207)	AWIN	Official training (AWIN)	Live	1	1 min	125	/
Czycholl et al. (208)	AWIN	Official training (AWIN)	Live	1	1 min	125	Term and PC level
Czycholl et al. (209)	AWIN	Official training (AWIN)	Live	1	1 min	125	PC level
Dai et al. (210)	FCP; own creation	Other training	Video	1	39–110 s	125	Term and PC level
Gronqvist et al. (68)	Minero et al. (220)	No training reported	Video	1	10 s	5-point scale	Term level
Harvey et al. (53)	Not reported	Other training	Video	1	1–252 s	Not reported	/
Jaramillo et al. (28)	FCP; own creation	Other training	Video	1	3 s	Not reported	Term and PC level
Minero et al. (211)	Literature; focus group; own creation	Other training	Live	1	1 min	125	PC level
Mullan et al. (212)	Not reported	No training reported	Live	1	Not reported	Not reported	PC level
Popescu et al. (213)	AWIN	Official training (AWIN)	Live	1	30–60 s	125	PC level
Rowland et al. (214)	Focus group; own creation	No training reported	Live	1	2–3 min	Not reported	PC level
Ruet et al. (215)	AWIN	Other training	Video	1	8 min	100	PC level
Ruet et al. (216)	AWIN	Other training	Live	1	1 min	125	Term level
Dai et al. (217)	AWIN	Other training	Live	Whole herd	2.5–10 min	125	PC level
Dai et al. (218)	Minero et al. (220) (AWIN)	Other training	Live	1	2.5–10 min	125	PC level
Gonzalez et al. (219)	Minero et al. (220) (AWIN); own creation	Other training	Video	1	1–450 s	Mercalli scale	Other
Minero et al. (220)	Literature; focus group; own creation	Other training	Live; Video	Whole group	7.5–15 min	125	PC level
Arena et al. (54)	Arena et al. (240) (FCP); literature; expert opinion	Other training	Video	Whole group	1.5 min	125	PC level
Arena et al. (55)	SQP	No training reported	Live	1–5	1 min	Not reported	/
Barnard et al. (221)	Arena et al. (240) (FCP); SQP	Other training	Video	1 - whole group	1.5	125	Term level
Berteselli et al. (222)	Arena et al. (240) (FCP); SQP	Official training (SQP)	Live	Whole group	1	125	Term level
Berteselli et al. (223)	SQP	No training reported	Not reported	Not reported	Not reported	Not reported	(Term and PC level)
Cuglovici and Amaral (224)	SQP	No training reported	Live	Whole group	1	125	Term level
Harvey et al. (225)	Arena et al. (54, 55); own creation	Other training	Video	1	30 s – 2 min	125	PC level
Menchetti et al. (31)	Literature; own creation	Official training (SQP)	Live	1	Approx. 80 s	5-point scale	Term and PC level
Pedersen and Malm (48)	Own creation (consulted QBA experts)	Other training	Live	1	15–45 min	125	Term level
Raudies et al. (226)	Not reported; SQP	Not reported	Live; Video	Not reported	Not reported	Not reported	/
Shaw et al. (227)	Arena et al. (54, 55); own creation	Other training	Video	1	2 min	125	PC level
Stubsjøen et al. (228)	Literature; focus group; own creation	Other training	Video	1–5	Not reported	125	Term and PC level
Stubsjøen et al. (229)	Stubsjøen et al. (228)	Other training	Live; Video	1 - whole group	2	125	Term and PC level
Heritier et al. (230)	SQP; literature; from FCP; focus group; own creation	/	Not reported	Not reported	Not reported	Not reported	Term level
Travnik and Sant’Anna (231)	Literature; own creation	Other training	Video	1	12 min	126	PC level
Travnik et al. (232)	Travnik et al. (232)	Other training	Video	1	12 min	Not reported	PC level
Jarvis et al. (233)	Field observations; focus group; own creation	No training reported	Live	1	60 min	125	PC level
Wiese et al. (44)	Literature; field observations; focus group; own creation	Other training	Video	(80)	1 min	100	PC level
Stagni et al. (234)	Literature; field observations; own creation	Other training	Live	1	20 min	125	Term and PC level
Delfour et al. (32)	Own creation	No training reported	Live	1 - group	Not reported	Not reported	/
Yon et al. (235)	Literature; field observations; own creation	No training reported	Live	1	1 min	125	Term and PC level
Dobrikj et al. (236)	Yon et al. (235)	No training reported	Live	1 - group	1 min	Likert scale	Term level
Gartland et al. (33)	Literature; own creation	No training reported	Live	1	Not reported	5-point scale	Term level
Munerato et al. (45)	Not reported	No training reported	Live	1	Not reported	125	Term and PC level
Skovlund et al. (237)	Literature; field observations; own creation	Other training	Live	1	5 min	125	PC level
Nogueira et al. (238)	Literature; own creation	No training reported	Video	Not reported	20 s	125	Term and PC level

VAS, Visual Analogue Scale; FCP, Free-Choice Profiling; WQ, Welfare Quality; AWIN, European Animal Welfare Indicators; SQP, Shelter Quality Protocol.

“/” Is provided when the not applicable (e.g., no results provided for QBA) or when the information was not possible to extract.

“According to WQ/AWIN” is provided when it was stated, or it could be elucidated, that the methods followed these protocols and the sampling guidelines (e.g., for WQ, VAS = 125 mm, no. of animals assessed = group).

1 “WQ,” “AWIN” or “SQP”: the list originates from these protocols; “Literature”: based on terms or lists collected in the literature; FCP: the list was generated through Free-Choice Profiling; focus group: the list was generated/adapted in a focus group; “own creation”: The list was developed or adapted as part of the study; a listed reference refers to the study (included in this paper) the list originates from.

2 Reported training of the QBA observers and identifiable details of such training.

3 Number of individuals covered by each assessment (group/herd-level, or a subset hereof, is stated when the number of individuals was not clear from the text). In some instances, it could be determined that, e.g., WQ guidelines were followed and hence “Sample og group” could be provided.

4 Indicates the time of a single QBA (assessment) (some assessments are repeated, e.g., across the day).

^*^Same study, including cattle and pig.

### 3.1 Aim of the studies and origin of QBA term lists

The aim of the studies was in most cases welfare assessment (144 papers), and QBA was often done as part of the WQ (112 papers) or AWIN protocols (14 papers) (Table 1). Seven studies did not use QBA as an indicator in the area of general welfare assessment but rather as a measure of temperament [e.g., Gois et al. (22)]. The remaining studies’ aims can be summed up as assessment of emotional state independent of general welfare assessment, for example as an evaluation of an animal’s emotional response to specific events or contexts (e.g., disease, sport events etc.).

The greater representation of studies using QBA as part of a welfare assessment protocol, is also reflected in the origin of the FL, since the term lists came from either the WQ or the AWIN protocol in 141 out of the 193 studies (see Table 2). However, in many cases the protocols were modified by adding one or several new terms [e.g., Andreasen et al. (23) and Sans et al. (24)], by reducing the number of terms overall [e.g., des Roches et al. (25) and da Silva et al. (26)] or, e.g., by exclusively using negative valenced terms (27). When the FL was not part of an existing welfare assessment protocol (identified as WQ, AWIN or SQP), list development can be categorised as being either based on the literature (terms are collected in the literature to form a list), FCP (a new list was created based on the FCP approach) or using focus groups (terms were generated in a focus group), and was often based on a combination of these. While the studies on cattle, pigs and poultry most often used standardised lists (typically from WQ, Supplementary Table 1), the case is different for goats and especially sheep (Supplementary Table 2). Although AWIN has developed lists for these species (13, 15), several of the identified studies reported using self-developed lists, and it was not always clearly described how the lists had been developed. In the studies providing details on how the lists were provided, the authors often specifically highlight the need for developing alternative lists for specific purposes [e.g., (28)] or with regard to translation issues when used in different geographical regions [e.g., (12)]. However, in general, another notable fact concerning studies using alternative lists is that altogether, these lists vary greatly in the number of terms, for example in small ruminants, some consist of just six (29) and others of 21 (30) terms. Considering the details of the FL used, please see Supplementary Tables 1–4.

### 3.2 Experimental procedure of use of QBA

Again, most of the studies applied the QBA according to the methodology described in the respective welfare assessment protocols. However, some differences can be detected, for example regarding the length of the VAS. Eight studies reported using a VAS of only 100 mm (instead of 125 mm as originally described in WQ, AWIN and SQP), and two studies reported a VAS of more than 125 mm. Further adaptations of the VAS were also found in the form of using, e.g., survey software formats, categorical or Mercalli scales [e.g., Menchetti et al. (31), Delfour et al. (32), and Gartland et al. (33)]. Noteworthy, three studies (34–36) used a novel method they termed continuous (c) QBA (c-QBA). C-QBA is a combination of QBA with the “Temporal Dominant Behavioural Expression” methodology (34) and enables recording shifts in individual QBA descriptors over time, i.e., the description of changes in animal behavioural expression during the observation session.

For the production animals, whole groups of animals were typically observed (following the WQ approach for these species), with only a few exceptions [e.g., Ebinghaus et al. (37, 38) and Gois et al. (22)]. For companion animals (including horses, but not donkeys) as well as for the zoo animals the reverse is true; most of the studies observed the animals at an individual level. The total number of animals included in the studies differed widely, with larger numbers of animals observed in production animals, with studies on hens and broilers including the highest numbers. The time frame observed per animal group was in most studies determined by the respective welfare assessment protocols, although different time frames also can be found (see Table 2).

Most of the studies observed the animals directly, while 29 studies used indirect (video) observation, and 11 studies used both direct and indirect observations. The level of observer training was found to vary greatly across the studies and was often not reported or was poorly described. Most studies provided no information on the level of experience with the relevant species (results not included in tables), while 89 studies reported their observers as experienced, however with large variation in provided details and in level of experience. Concerning the number of observers that performed QBA, 29 studies did not provide details, 49 studies were based on observations by one observer, 24 on observations by two observers and the remaining studies on observations by multiple observers. However, of the studies using more than one observer, only 43 reported that observer agreement was checked before data collection. A large variation is found in how observers were trained and how agreement was reached, checked and reported. This ranges from reporting of simple discussions about terms among observers, reaching an overall consensus of the whole WQ, AWIN or SQP (of which QBA is part), to utilising a few videos or spending up to multiple days or weeks on on-site training. Likewise, the analysis of observer agreement varied from descriptive evaluations to different statistical analyses, in which the level of interpretation also varied.

### 3.3 Reported statistical analysis

As shown in Table 2, 20 studies analysed the results of QBA outcomes solely on term level, 56 studies used the aggregation system of WQ, and 93 studies used a PCA for analysis (these are reported on in more detail in Supplementary Table 5). Fifteen of these 93 studies provided information on data suitability criteria. Fifty-two of the studies retained two PCs to explain the outcomes of the QBA (as in the WQ protocol), the other studies either retained one component (two studies), three components (20 studies, with 17 studies interpreting the third extracted component further) or four components (eight studies, with five studies interpreting the third and fourth extracted principal component further). In less than half of these 93 studies, information on cut-off values for factor loadings that were used for interpretation of the respective components could be extracted, i.e., most of the included studies did not state what was interpreted as loading highly on a PC and thus which values were used for interpreting/naming a PC. In some cases, this information was not clearly reported in the material and methods section, but could be extracted from the results tables. In about a quarter of the studies, principal component loadings of above 0.4 and below −0.4 were reported as used as cut-offs for this interpretation.

## 4 Discussion

Overall, FL QBA has been used in a variety of species, in many different settings and contexts, with various approaches to its methodology and analysis. The majority of FL QBA studies were carried out as part of a welfare assessment protocol for farm animals. While a large variation in species is evident, the literature search yielded no results on experimental animals. This is somewhat surprising, as the method aligns with other qualitative approaches used in experimental animals, such as those included in some forms of pain grimace assessments [e.g., in rabbits: Benato et al. (39)]. Moreover, using the method in experimental animals may aid in substantiating the validity and reliability of QBA, as laboratory settings typically offer more controlled environments [e.g., Calisi and Bentley (40) compared to, e.g., on farm or in zoos]. Overall, there is a variation in a number of factors that are likely to affect the outcome of a QBA and its meaning. Differences in the conditions under which they were observed (e.g., filmed or live), choice of terms in FL and statistical analysis, makes it difficult to compare the results of the current studies even on the species-level.

### 4.1 Aim of the studies

The original development of QBA was aimed at the evaluation of welfare (4, 5, 20), arguing that its whole-animal expressive information could make a unique contribution to scientific welfare assessment. Multiple validity and reliability studies on the FCP approach were carried out resulting in a generally proven efficacy of the methodology [reviewed by Wemelsfelder (8)]. Likewise, the high feasibility owing to its rapid assessment and ease of implementation [e.g., (9–11)], compared to other methods of assessing the emotional state [such as the cognitive bias test; Crump et al. (41)] is a clear advantage of QBA. These advantages likely contribute to QBA being included as a customary part of various welfare assessment protocols. The first FL developments were specifically carried out for inclusion in welfare assessment protocols for farm animals (9–11). With this development, it is not surprising that the far most common use of FL QBA in the included studies was identified as general welfare assessment, and predominantly as part of the established frameworks of WQ, AWIN and SQP, belonging to the welfare criterion ‘positive emotional state’ [e.g., Botreau et al. (42)]. Also outside such larger protocols, and following some concern for QBA being at risk for subjectivity due to the reliance on human observers (43), it is generally recommended not to use Fl QBA as a stand-alone indicator for welfare assessment but to combine and cross-validate it with other indicators, as for example Andreasen et al. (23) could not validate QBA as stand-alone indicator for welfare assessment.

Despite this focus on general welfare assessment, the FL QBA has by now been used for a variety of aims. In fact, the second-most common use was its application in specific contexts, mainly to assess emotional reactions to certain events, such as intrusive sampling and capture of, e.g., salmon (44) and pampas deer (45), calf-roping events during rodeo (46), agonistic social encounters in pigs (47), dogs’ interaction with humans during canine-assisted interventions (48) and sport events (28, 49). In these studies, FL QBA was mainly applied to investigate potential impacts of such events on animals’ emotional states and how this might affect their welfare. Further aims included temperament assessment. In general, the various aims showcase a broad and flexible usage of the method within the context of assessment of emotional state as also suggested by Boissy et al. (1). Therein, it should specifically be noted that in comparison to other methods of assessment of emotional state, QBA takes the whole-body language into account (4) instead of relying on separately measuring specific mimics, gestures or body postures [e.g., ear position, play and all grooming in cattle (2)].

### 4.2 Origin of QBA lists

Because the descriptors that constituted the lists developed for WQ and AWIN were not necessarily appropriate or optimal for other types of situation and contexts, alternative lists were developed for other purposes such as the study of sick animals (25), human-animal relationship tests (37, 50), mother-young interactions (51) and sport competitions (in contrast to the evaluation in the normal husbandry environment) (28). Moreover, it should be noted that translation and cultural interpretation issues might arise concerning the descriptors which might make development of lists for use in specific geographical regions necessary as highlighted by Souza et al. (12). These different circumstances, as well as different aims other than welfare assessment, justify the use of different lists. There was however, some variation in how the FL were developed. However, not all these lists were developed as originally described by a first step of FCP, and the creation of a validated FL as well as the process of FL development or justification of selection of terms used was not always clearly described [e.g., Carroll et al. (52), Kaurivi et al. (27), and Harvey et al. (53)]. It should be noted that many of these studies detailing on development and validation of FL specifically pointed out that the terms included should cover many different affective states and the reduction of terms without any further validation is thus not recommended [e.g., Arena et al. (54, 55) and Souza et al. (12)]. Therewith, there is in principle also a minimum number of terms that should be used in QBA. This study presents an overview of all FL that have been used to date, however, as pointed out, the level of validation of these lists varies.

### 4.3 Experimental procedure of use of QBA

Most of the studies used a VAS of 125 mm in length. In the first mention in Wemelsfelder et al. (4) of a VAS in the context with QBA, a length of 12.5 cm was described. Since then, and especially in the first developments of FL QBA for welfare assessment protocols (9–11), this length was most commonly used. The authors of this study are not aware of any justification for using 12.5 cm in QBA to have been reported in literature. In human medicine, the most common length of used VAS is 10 cm (56), which also is the second most common VAS length identified in the present literature review. In a controlled trial on patients’ VAS preferences, Sriwatanakul et al. (239) found 10 cm to be the length of the most preferred type of VAS. Another study by Seymour et al. (58) on specifically comparing different VAS lengths, reported a 10 cm continuous scale as the most appropriate, and in general, that lengths from 10 to 15 cm were suitable. Consequently, it is not clear whether 100 mm and 125 mm differ in suitability. The authors of the present study are not aware of any studies that investigate potential effects of VAS length on QBA outcomes. Such knowledge might be beneficial in order to unify the QBA methodology and for comparability across QBA studies.

In addition to the differences in VAS lengths, a few alternative measurement techniques used for QBA were identified: for example, Gartland et al. (33) rated various gorilla expressions and activity patterns on qualitative descriptors such as anxiety, curiosity, irritability, cooperation and dominance, using a categorical 1–5 scale (ranging from ‘very low’ to ‘very high’). Moreover, in some articles, the method referred to as ‘continuous QBA’ (c-QBA) was introduced. C-QBA works with individual descriptors and is based on temporal dominance of sensations (TDS) procedure, which allow raters to detect behavioural fluctuations *during* sessions, as opposed to the classical approach of QBA where the sum of behavioural expression is considered and rated *after* a session. C-QBA hence provides information on variation over time in discrete emotions, i.e., shifts in behavioural expressivity over time can be captured. C-QBA was developed for goats (35) and buffaloes (34, 36). These approaches use the same type of qualitative descriptors as QBA, based on whole-animal expressive demeanour, and so require the same type of observational assessment and therefore were included in this review. However, in contrast to the original QBA, the format in which such assessments are subsequently processed and analysed differs.

Large variation was identified in the experimental setups across studies, which is not surprising given the general variation of the purpose and context of the studies. Hence, some studies observed groups of animals while others focused on individuals. Moreover, the size of the group under observation varied largely and was not always clearly reported on. This depends naturally on the species being studied and feasibility in the settings (i.e., groups are more likely to be observed in production animals, explainable by the husbandry environment on farm). However, at this stage, it remains unclear as to what effect individual vs. group-level observations has on FL QBA outcomes. To the authors’ knowledge, this has not yet been investigated. Likewise, in the majority of the studies, direct observations, rather than video-based observations, were used. It is plausible that video observations may yield more accurate results and improved observer focus, since there may be less external disturbances (59–61). On the other hand, assessors may be less involved, meaning that their actual ability to integrate perceived details of behaviours and context and transfer that into descriptors may be limited due to not all information being transferred and the observers are also less able to react to, e.g., sudden changes on-site (which they might not even be aware of) (62–64). The question such arises on whether observers should be informed about the context or background or not when using video observations. A general disadvantage of video observation is moreover the additional costs and time involved (64) that should be taken into account with regard to feasibility of the assessment. Cooke et al. (65) investigated the difference between direct and video observations of beef cattle, and found no difference between the methods for PC1, whereas the response was less pronounced for PC2 for the video observations. Consequently, the authors of the study did not recommend using video observations for QBA. In contrast, Czycholl et al. (62) found good reliability for QBA when based on video observations, but not for on-farm assessments. A possible explanation for the difference in results is that in the study by Cooke et al. (65), the observers were in both cases looking at the same animals, whereas in Czycholl et al. (62), the live observations were carried out in the same section of the farms, however not necessarily on the same animals.

The results of this review further show a large variability in the level of observer training and experience. Tuyttens et al. (66) focused their study on observer bias and effects of observer training and proved an influence on both quantitative and qualitative methods (specifically also the QBA). Likewise, in a QBA-like study Meyer et al. (67) suggested that there were possible interactions between observer experience with dogs, and interpretation of dog behaviour (amongst others). Furthermore, Gronqvist et al. (68) highlighted the importance of experience with a species to correctly interpret potentially dangerous situations and Broom and Johnson (69) emphasised that knowledge about the behaviour of a species is important to avoid misinterpretations. Likewise, in the initial introduction of FL QBAs for welfare assessment for cattle, pigs and poultry alike, it was mentioned that for use of FL QBA, observers need to be trained and experienced (9–11). Accordingly, the most common welfare assessment protocols all highlight the need for a sufficient training level of observers before using welfare assessment protocols (of which QBA is part of). On the contrary, the first introductory publication on FCP QBA worked with observers naive to the species, relying on the general ability of humans to assess the qualitative body language signals (5). Although in principle, QBA methodology thus can work with naive observers, overall, like in quantitative behavioural observations, species experience and training of observers can improve the reliability. Guidelines regarding training level and requirements of species experience when using FL QBA could be helpful with regard to a unification of the literature and thus an enhanced comparability and therewith enable the possibility of drawing better conclusions about the reliability and validity of the methodology and potential influence on results by specific settings.

### 4.4 Reported statistical analysis

Three main statistical approaches for retrieving FL QBA outcomes were identified: (1) utilising mm values on term level, (2) subjecting the QBA scores to a PCA and (3) aggregation following the WQ approach, based on expert opinion and pre-existing data. The latter was usually applied when the FL QBA was used as part of an existing WQ protocol. It should be noted that in the very first publications on QBA (which are carried out as FCP), statistical analysis was carried out by Generalised Procrustis Analysis (GPA) prior to PCA (4, 5). In the first publications concerning the development of a FL approach in the bounds of the WQ project, results were analysed on term level as well as by PCA (9–11). However, the respective authors argued that a PCA may be the most suitable approach to analysing QBA. Identifying principal components (PCs) on which the terms have a certain loading may help in a more valid interpretation with regard to, e.g., observer agreement. Thus, although the analysis based on term level was presented in those studies (9–11), the authors argued that conducting a PCA would provide more reliable results. This would mean that the analysis based on term level, which occurred in 20 studies cannot be seen as the most appropriate analysis.

Regarding PCA, it should be noted that the data needs to meet certain prerequisites such as certain sample size requirements and interval-level measurement for this statistical method to be applied. In textbooks, it is described that each PC should at least have an eigenvalue of >1 (Kaiser-Guttman criterion), a clear break in eigenvalues is seen between the PCs (scree-test) and a certain amount of variance of the data set is actually explained by the extracted PCs. Additionally, there is the interpretability criterion with regard to variables loading highly on the extracted PCs (70, 71). A further useful parameter to assess the data suitability is the Kaiser-Meyer-Olkin criterion, which was actually invented for factor analysis (72, 73). Looking at the results of this review, it becomes clear that only 15 of 93 studies using PCA as analysis actually tested their data for data suitability criteria beforehand. In textbooks regarding the PCA, factor loadings to be interpreted as meaningful are named as >0.4 (70) or even higher (>0.6–0.7) (74, 75). Looking at the results of this review, 19 of the studies using PCA interpreting factor loadings used cut-off values of >0.4 and three studies used cut-off values of >0.6. It is probably a matter of study aims whether the use of clear cut-off values of factor loadings or the pattern of loadings showing which descriptors contribute most to the identified PCs (70) is most suitable and how informative relatively low-value loadings then are. That said, the use of term-loadings close to zero for interpreting a PC should always be treated with caution. However, using clear cut-off values might be impossible, as, depending on the exact model used (and different statistic programmes use different models by default), exact values may differ. This is further complicated by the fact that some authors [and in some cases it may be justifiable (70)] also use a factor analysis, but interpret it as a PCA. Another noteworthy result of the present literature review is that most of the studies that conducted a PCA extracted two PCs and interpreted those further without explicit reference to the use of general rules for extraction such as scree plot or Guttman criterion (70). This may be due to the fact that studies started adapting their methodology to that of other studies without adjusting it to their own data, which is a known risk and phenomenon in science (76). Not meeting the prerequisites of data for statistical data analysis, incorrect extraction or over interpretation of relatively low values includes quite clearly the risk of misinterpretations (70). This all being said, PCA is in general a relative flexible method and over the years has been adapted to a variety of disciplines (77), so it seems well-suited also for analysis of QBA. However, the findings described highlight the need for more advice on how to correctly use and interpret multi-variate statistical techniques such as PCA for the analysis of QBA data.

The third way of statistical analysis found is via the aggregation system suggested and published by WQ (in the case of FL QBA being a part of a larger welfare assessment protocol). This aggregation system has the general aim of aggregating all the different welfare indicators (of which the FL QBA is only one) into one final welfare score of 0–100 (78). While in total, many different methods for aggregation are used, for the FL QBA, basically weighted sums are used which were obtained by expert opinion and PCA on—by nature of the studies—limited data sets that had conducted FL QBAs (9–11, 42). Those data sets were also limited to certain regions [e.g., 17 farms in Germany, three assessors: (9)], the results obtained from the PCAs on these limited data sets may not be generalizable. It is a well-known fact that small study populations easily lead to over- and under-estimations (79). A solution to overcome this in the future, due to the points raised above, would be a revision of the existing aggregation system, e.g., by joint use of the now available larger data sets of, e.g., WQ data from different working groups and countries, which also aligns with the general aim of many welfare assessment protocols (e.g., WQ) to enhance and revise the existing protocol as new knowledge arises (80).

### 4.5 Quality of the literature search

The high interobserver reliability between the extractors, along with the fact that only studies after 2011 were extracted [whereby the first developments of FL QBA are described in the Welfare Quality Reports in 2009 (9–11)] and that the last search without species specific terms did not result in any further articles, demonstrates the quality of this literature review.

## 5 Conclusion

In conclusion, the FL QBA approach has been used across many species, primarily farm animals, but also companion animals and more recently also zoo animals. However, there was during this time no QBA developed for experimental animals. FL QBA has been used for a variety of aims, however mainly for evaluating emotional state and most often as part of welfare assessment, which is also what the FL QBA was originally developed for. Different aims and settings will call for specifically tailored FL in order to strengthen reliability and validity of QBA in those settings. However, if a FL must be standardised as part of larger welfare assessment protocols, then it is advisable to clarify the context in which that list of terms can be used. A number of methodological aspects of FL QBA vary in the identified studies, ranging from using different lengths of VAS, to the evaluation of animals at group/individual level, time used for observation, training and experience level of observers and several other factors. Moreover, different statistical analyses are used, and it is identified that not always respective prerequisites for the use of those methods exist. These are aspects to consider when gathering knowledge of the current level of reliability and validity of QBA. Future studies should thus address the question whether or not there are certain conditions that must be met when applying QBA and what conditions these are, taking into account that studies have different aims and are applied in different settings, which may require a certain flexibility in using and interpreting QBA. This could answer the question whether clearer guidelines on the construction, use and statistical analysis of FL QBA are necessary and allow the potential development of such. This could then also include guidance on how and which results on FL QBA need be presented to encourage cross-study comparison.
