# Supplementary material for: Literature review of the use of Qualitative Behaviour Assessment with a fixed list of terms
Source: Front Vet Sci. 2026 Jan 9;12:1588346. doi: 10.3389/fvets.2025.1588346 (PMC12827162; doi:10.3389/fvets.2025.1588346)
Supplement: Supplementary file 5 [file Table_5.DOCX]

**Supplementary Table 5.** Overview of statistical parameters as reported by the authors in the identified studies, including methods to assess data suitability for PCA, the number of PCs retained and labelling for interpreting QBA outcomes, as well as the reported term factor loadings used for PC interpretation.

| **Reference** | **Method of data suitablity^1^** | **No. of retained PCs^2^** | **Variance explained by PCs (%)^3^** | **PC1 name^4^** | **PC2 name** | **PC3 name** | **PC4 name** | **Factor loadings  (cut-off/reported)^5^** |
| --- | --- | --- | --- | --- | --- | --- | --- | --- |
| Andreasen et al., 2013 | Not reported | 2 | 56 (44 + 12) | Calm/relaxed to uneasy/agitated | Indifferent/distressed to lively/playful | / | / | 0.24/-0.24 |
| Andreasen et al., 2020* | Not reported | / | / | / | / | / | / | / |
| Brscic et al., 2019 | Not reported | / | 54 (40.3 + 13.3) | Valence | Arousal | / | / | 0.48/-0.36 |
| Ceballos et al., 2021 | Not reported | 3 | 61.27 (33.2 + 16.6 + 11.4) | Responsiveness | Alertness | Defensive aggression | / | 0.6 |
| Collins et al., 2021a^6^ | / | / | / | / | / | / | / | / |
| Cooke et al., 2022 | Not reported | 3 | 60.6 (27.8 + 21.4 + 11.4) | Arousal | Mood | Alertness | / | Not reported |
| Cooke et al., 2023 | Not reported | 2 | 49 (27.81 + 19.01) | Arousal | Mood | / | / | Not reported |
| de Boyer des Roches 2018 | Not reported | 2 | 58 (35 + 23) | Active/happy to suffering/lethargic | Fearful/tense to confident/calm | / | / | 0.6 |
| Ebinghaus et al., 2016 | Not reported | 2 | 85 (78.6 + 6.4) | Relaxation/attraction/trust to fear/stress/aversiveness | / | / | / | Not reported |
| Ebinghaus et al., 2017 | Not reported | 2 | 74.9 (67.5 + 7.4) | Relaxation/trust to fear/aversion | / | / | / | Not reported |
| Ebinghaus et al., 2018 | Not reported | 2 | 67.5 for PC1 (PC2 not reported) | Relaxation/trust to fear/aversion | / | / | / | 0.6 for PC1, 0.4 for PC2 |
| Ebinghaus et al., 2020 | Not reported | 2 | 67.5 for PC1 (PC2 not reported) | Relaxation/trust to fear/aversion | / | / | / | / |
| Ebinghaus et al., 2022 | Not reported | 4 | 83 (49.9 + 18.1 + 8.9 + 6.4) | (Valence) | (Arousal) | / | / | Not reported |
| Ellingsen et al., 2014 | KMO | 4 | 84.9 (25.5 + 24.4 + 18.1 16.9) | Calm/patient | Dominating/aggressive | Positive interactions | Insecure/nervous | 0.30 |
| Gois et al., 2016 | Not reported | 2 | 54, 45.4, 45.5 (three farms, PC1); 15, 15.6, 15.6 (three farms, PC2) | Temperament index | / | / | / | 0.75 (contribution to PC) |
| Gutmann et al., 2015 | KMO | 2 | 53 (35 + 18) | Mood | Activity | / | / | 0.6 |
| Rizzuto et al., 2020 | Not reported, factor analysis | 4 | 72 | Negative emotions (round 1 and 2) | Positive emotions (round 1); neutral emotions (round 2) | Neutral emotions (round 1); positive emotions (round 2) | / | Not reported |
| Russell et al., 2023  **Table 5.** (*continued*) | Centred and normalised scores | 2 | 54.2 (38.45 + 15.7) (group 1); 57.42 (40.03 + 17.39) (group 2) | Content/relaxed to fearful/bored (group 1); bored/fearful to content/relaxed (group 2) | Lively/playful to pathetic/bored (group 1); lively/inquisitive to bored/apathetic (group 2) | / | / | 0.67/-0.39 |
| Sant'Anna & da Costa, 2013 | Not reported | 2 | 59 (49.47 + 10.21) | Agitated/active to calm relaxed | Irritated/curious to attentive/fearful | / | / | Not reported |
| Santos et al., 2020 | Not reported | 2 | 67 | Positive body expressions | Negative body expressions | / | / | Not reported |
| Schmitz et al., 2020 | Not reported | 1 | 67.8 | Relaxation/trust to fear/distress | / | / | / | Not reported |
| da Silva et al., 2021 | (Reductionist analysis) | 3 | 73.32 (55.85 + 17.47 + 6.94) | Scared - irritable; nervous - aggressive; distressed - restless; confused - fearful; bored - anxious; curious to contented, calm - even-tempered; sociable - friendly; happy - lively | / | / | / | Not reported |
| Camerlink et al., 2016 | Note reported | 2 | 59 (43.3 + 15.7) | Valence | Arousal | / | / | 0.3/0.5 |
| Cardona et al., 2022 | Tests for normality, homoscedasticity (visually) | 2 | 66.12 (44.45 + 21.67) | Positive emotions index | Negative emotions index | / | / | 0.6 |
| Cardona et al., 2023 | Not reported | 3 | 72.4 (46.68 + 16.64 + 9.08) | Positive high arousal index | Negative high arousal index | Positive low arousal index | / | 0.6 |
| Carroll et al., 2018 | KMO | 2 | 80.9 (69.4 + 11.5) | Good welfare | Aggressive | / | / | 0.3 (but all terms 0.4/-0.4) |
| Clarke et al., 2016  **Table 5.** (*continued*) | Box-Cox transformation to achieve normal distribution | 4 | 75.14 (37.58 + 17.89 + 14.03 + 5.64) | Calm/relaxed/content to tense/agitated/irritable | (Interpretation not straight forward; captured differences in use VAS among observers) | Bored/ listless/ aimless to active/lively/sociable | Enjoying/ happy to sociable/ playful | 0.32 (>75% of the highest absolute correlation coefficient value) |
| Czycholl et al., 2017a | Not reported | 2 | 42 - 75 | / | / | / | / | Not reported |
| Duijvesteijn et al., 2014 | Not reported | 2 | 19 - 55 (depending on stakeholder groups) | Depending on stakeholder group: Happy, satisfied, enjoying to frustrated, irritated, tense/distressed, indifferent, listless | Depending on stakeholder group: Active, lively/tense, frustrated, irritable | / | / | Not reported |
| Friedrich et al., 2019 | Not reported | 2 | 67.3 - 75.6 (three separate comparisons) | / | / | / | / | Not reported |
| Friedrich et al., 2020a | Not reported | 2 | 68.6 (observer 1); 74.4 (observer 2) | / | / | / | / | Not reported |
| Friedrich et al., 2021^6^ | / | / | / | / | / | / | / | / |
| Martinez et al., 2022 | Not reported | 3 | 79 (34 + 24 + 21) | Emotional balance/social interactions | Energy level | Focused activity of reacting with environment | / | Not reported |
| Meyer-Hamme et al., 2018 | Bartlett's Test, KMO | 2 | 60.4 (39.4 + 21.0); 58.1 (35.3 + 22.8); 56.3 (32.4 + 23.9) | Active behaviour with positive connotation-inactivity | Negative behaviour - positive inactivity | / | / | 0.4 |
| Munsterhjelm et al., 2015a | Low extraction communality (h²) + Cronbach’s alpha, Bartlett's test, KMO | 3 | 63.9 (23.6 + 22.4 + 17.9) | Fighting | Lack of bedding | Disease | / | 0.3 |
| Munsterhjelm et al., 2015a | Low extraction communality (h²), Cronbach's alpha, Bartlett's test, KMO | 3 | 74.8 (33 + 22.5 + 19.3) | Active positive behaviour | Passive negative behaviour | Passive positive behaviour | / | 0.5 |
| Munsterhjelm et al., 2015a  **Table 5.** (*continued*) | Low extraction communality (h²), Cronbach's alpha, Bartlett's test, KMO | 3 | 78.6 (30.7 + 28.4 + 19.5) | Active positive behaviour | Passive negative behaviour | Passive positive behaviour | / | 0.5 |
| Munsterhjelm et al., 2015a | Low extraction communality (h²), Cronbach's alpha, Bartlett's test, KMO | 3 | 64.2 (22.2 + 22 + 20) | Lack of bedding | Lack of resources | Lack of fibre | / | 0.3 |
| Munsterhjelm et al., 2015b | Not reported | 3 | 74.8 (33 + 22.5 + 19.3) / 78.6 (30.7 + 28.4 + 19.5)  (data from Munsterhjelm et al., 2015a) | Active positive | Passive negative | Passive positive | / | 0.5 (data from Munsterhjelm et al., 2015a) |
| Munsterhjelm et al., 2015b | Not reported | 3 | Not reported (data from Munsterhjelm et al., 2015a) | Active positive | Passive negative | Passive positive | / | 0.5 (data from Munsterhjelm et al., 2015a) |
| Oldham et al., 2021 | Not reported | 3 | 68 (32 + 20 + 16) | Agitated/tense to relaxed/content | Fearful/aimless to enjoying/confident | Listless/indifferent | / | 0.3 |
| Schmitt et al., 2019a | Not reported | 2 | 48 (33 + 15) | Positive feelings | Low arousal | / | / | Below 0.3 |
| Schmitt et al., 2019b | Not reported | 2 | 50.3 / 55.5 (33.6 / 39.2 + 16.7 / 16.3) | Lively, enjoying, content, happy, relaxed, calm to fearful, tense, distressed / content, playful, happy, calm, enjoying to tense, frustrated | Bored, positively occupied, sociable, playful, happy to indifferent, calm / relaxed, aimless, listless, bored to indifferent, active, fearful | / | / | (Eigenvector value above or below 0.25) |
| Temple et al., 2011b | Not reported | 2 | 58 (42 / 16), 59 (43 / 16), 59 (37 / 22) | Mood | (Interpretation not straight forward) | / | / | Not reported |
| Temple et al., 2013 | Not reported | 2 | 52 (35 + 17) | Mood | Arousal | / | / | Below 0.3 |
| Vitali et al., 2020  **Table 5.** (*continued*) | / | 2 | 50.7 (28.7 + 22.0) | Valence | Arousal | / | / | Not reported |
| Vitali et al., 2021 | Not reported | 2 | 71.9 (47.6 + 24.3) | (Observation day) | (Building) | / | / | Not reported |
| Bassler et al., 2013 | / | 2 | 43 (25 + 18) | Calm/ relaxed/content to agitated/tense/nervous | Content/energetic/confident to helpless/drowsy/ bored | / | / | 0.21 |
| Muri et al., 2019 | Not reported | 2 | 70.4 (48.3 + 22.1) | Arousal | Mood | / | / | Not reported |
| Sans et al., 2021b | Not reported | 2 | 54.34 (28.18 + 26.16) | Mood | Distressed to aggressive | / | / | Not reported |
| Sans et al., 2021a | Not reported | 4 | 66.2 (23 + 20.7 + 12.1 + 10.4) | Agitated/apprehensive/scared/distressed to calm/relaxed/tranquil/comfortable | Inquisitive/confident/aggressive/interested/lively/positively occupied to apathetic/dull/lethargic/disturbed/ fearful/distressed | Interested/confident/inquisitive/frustrated/aggressive/painful/playful | Active/agitated/positively occupied/comfortable/playful/inquisitive/confident/interested/frustrated | Not reported |
| Sans et al., 2023 | Not reported | 2 | 72.5 (45.4 + 27.1) | Emotional state (comfortable/friendly/relaced/confident satisfied to frustrated/unsure) | Energetic/active agitated/tense/nervous/scared to bored/frustrated/distress/drowsy | / | / | Not reported |
| Souza et al., 2015 | / | / | / | / | / | / | / | / |
| Souza et al., 2021 | Not reported | 4 | 69.4 (36.8 + 18.2 + 9.4 + 5.0) | Disturbed/frustrated to comfortable/lively | Calm/dull to agitated/active | Apathetic/relaxed to active/painful | Desperate/comfortable to dull/apathetic | Not reported (as low as -0.051) |
| Vasdal et al., 2022 | Not reported | 3 | 82.5 (50.8 + 19.7 + 12.0) | Mood | Alertness | Arousal | / | Not reported |
| Bodas et al., 2021 | Not reported | 2 | 43 (28 + 15) | Not reported | Not reported | / | / | Not reported |
| Collins et al., 2021b | Not reported | 2 | 30.5 + 24.5 | Valence | Arousal | / | / | -0.61 to 0.79 (>75% of highest absolute coefficient)) |
| Diaz-Lundahl et al., 2019 | Not reported | 2 | >60 (44.5 + 18 / 16.6) | Mood | Arousal | / | / | Not reported |
| Mialon et al., 2021  **Table 5.** (*continued*) | KMO | 2 | 61.2 (39.9 + 21.3) | Activity | Perturbation | / | / | Not reported |
| Muri and Stubsjøen 2017 | Not reported | 2 | Part1: (40.9 + 15.8); Part2: (31.2 + 21.3) | Not reported | Not reported | / | / | Not reported |
| Hernandez et al., 2020 | Not reported | 2 | 82 (67 + 14) | Valence | Energy level | / | / | Not reported |
| Phythian et al., 2013 | Not reported | 2 | 80 (49 + 31); 80 (52 + 28); 81 (48 + 33) | Positive/negative experience | Arousal | / | / | Not reported |
| Phythian et al., 2016 | Not reported | 2 | 68 (47 + 21) | Mood | Responsiveness | / | / | Not reported |
| Stubsjøen et al., 2022b | Not reported | 2 | 50 (34 + 16) | Mood | Not interpreted further | / | / | Not reported (mentions strongest loadings of min. 0.33) |
| Battini et al., 2016 | Not reported | 2 | 49.04 (31.27 + 17.77) | Emotional state | Activity | / | / | Not reported |
| Battini et al., 2018 | Not reported | 4 | 62.43 (27.16 + 14.75 + 10.55 + 9.96) | At ease with the environment | Emotions connected to the quality of the environment and the possibility to interact with it | / | / | Not reported |
| Battini et al., 2021 | Not reported | 2 | 62.5 (42.04 + 20.53) | Valence | Arousal | / | / | Not reported |
| Can et al., 2016 | Not reported | 2 | 46 (28 + 18) | Emotional state | Activity level | / | / | Not reported (mentions strongest loadings of min. 0.52) |
| Grosso et al., 2016 | Not reported | 3 | 60.87 (29.04 + 19.70 + 12.13) | Mood | Arousal | Level of sociable interaction | / | 0.6 |
| Czycholl et al., 2019 | Not reported | 2 | 50 (37 + 13) / 56 (43 + 13) | Valence | (Interpretation not straight forward) | / | / | 0.4 |
| Czycholl et al., 2021 | Not reported | 2 | Not reported | Valence | (Interpretation not straight forward) | / | / | 0.4 |
| Dai et al., 2022 | Not reported | 3 (2 interpreted) | 72.4 (59.8 + 12.6 + 7.5) | Valence | Arousal | Not further interpreted | / | 0.2 |
| Jaramillo et al., 2023 | Horne's parallel analysis | 2 | 74.41 | Negative polarity | Positive polarity | / | / | 0.4/-0.4 |
| Minero et al., 2018 | Mardia's test (assumptions not met); Box's M test | 3 (2 interpreted) | 74 (48 + 17 + 9); 65.72 (39.1 + 16.5 + 10.2) | Valence | Arousal | Happy/looking for contact | / | (0.48) |
| Mullan et al., 2014  **Table 5.** (*continued*) | Not reported | 4 (2 interpreted) | 68.1 | Arousal | Mood | / | / | -0.025 |
| Popescu et al., 2022 | Not reported | 3 | Not reported | Negative to positive emotional states fearful/alarmed to friendly/ relaxed (first assessment); fearful/alarmed to friendly/relaxed/curious (second assessment; fearful/alarmed to friendly/relaxed/curious (third assessment) | Pushy to apathetic (first assessment); aggressive to uneasy/pushy (second assessment); aggressive to pushy (third assessment) | Apathetic to happy (first, second and third assessment) | / | 0.38 |
| Rowland et al., 2022 | Not reported | 2 | 56.9 (43.1 + 13.8) | Mood | Energy | / | / | 0.37 |
| Ruet et al., 2020 | Not reported | 2 | 57.1 (38.2 + 18.9) | At ease/relaxed | Alarmed/annoyed/pushy | / | / | 0.4 |
| Dai et al., 2016 | Not reported | 3 | 67.31 (32.56 + 19.05 + 15.7) | Arousal | Emotional state | / | / | Not reported |
| Dai et al., 2018 | Not reported | 3 (2 interpreted) | 69.1 (30.5 + 24.8 (+ 13.8) | Emotional state | (Interpretation not straight forward) | Not named/further interpreted | / | Not reported |
| Minero et al., 2016 | Not reported | 3 | 78.09 (43.7 + 22.49 + 12.80) | Mood | Arousal | Anxious/withdrawn to playful | / | Not reported |
| Arena et al., 2019a | KMO, anti-correlation matrix value | 4 | 70.9 (28.3 + 25.9 + 8.9 + 7.8) | Interest in environment (including people/other dogs) | Comfort vs. anxiety | Fearful/hesitant/wary | Depressed/bored | 0.6 |
| Harvey et al., 2019 | Not reported | 2 | 40.8 (25.3 + 15.5) | Stressed/anxious to comfortable/relaxed | Interested/explorative to bored/depressed | / | / | Not reported |
| Menchetti et al., 2019 | Not reported | 1 | Not reported | Aggressiveness sociability axis | / | / | / | Not reported (reports only loadings of 0.40 or -0.40) |
| Shaw et al., 2022  **Table 5.** (*continued*) | KMO | 4 (2 interpreted) | 80/81 | Overarching positive engagement | Arousal | Frustrated | Frustrated frustrated/anxious to sociable | 0.5/-0.5 |
| Stubsjøen et al., 2020 | Not reported | 2 | Study 1: 55.8 (33.5 + 22.3); Study 2: 56 (34.5 + 21.5) | Study 1: Indifferent, depressed, uncomfortable and bored to curious, energetic, sociable and expectant; Study 2: Depressed, indifferent, uncomfortable and bored to expectant, energetic, curious and sociable | Study 1: Relaxed, content and trustful to nervous, stressed, restless and aggressive; Study 2: Relaxed, content and indifferent to nervous, stressed, restless and frustrated | / | / | Not reported |
| Stubsjøen et al., 2022a | Not reported | 2 | 56.8 (34.9 + 21.9), 50 (30 + 20) | Mood | Arousal | / | / | Not reported |
| Berteselli et al., 2022 | / | / | / | / | / | / | / | / |
| Travnik and SantAnna 2021 | Not reported | 3 | 76.63 | Valence | Arousal | Aggressiveness/caution axis | / | 0.6 |
| Travnik et al., 2022 | Not reported | 3 | 66.93 (43.29 + 17.13 + 6.51) | Valence | Arousal | Aggressiveness | / | 0.6 |
| Jarvis et al., 2021 | Not reported | 4 | 79 (56 + 12 + 6 + 5) | Mood/energy | Listlessness | Association between listlessness and crowded conditions | Contrast between inquisitiveness and fear | Not reported |
| Wiese et al., 2023 | Not reported | 4 | 74.5 (36.7 + 17.7) | Relaxed/content/positive active to unsettled/stressed/spooked/skittish/agitated | Relaxed to energetic/purposeful/inquisitive | / | / | 0.166 (highlights strongest loadings) |
| Stagni et al., 2022 | KMO | 4 | 74.5 (43.8 + 12.9 + 10.9 + 7) | Mood | Activity levels | Emotions of joy and suffering | Frustration | Not reported |
| Yon et al., 2019 | Not reported | 1 | Not reported | At ease in the environment | / | / | / | Not reported |
| Munerato et al., 2015 | Not reported | 2 | 56.8 (37.3 + 19.5) | Suspicious/alert to calm/indifferent/sociable | / | / | / | Not reported |
| Skovlund et al., 2023 | KMO; Bartlett's test | 2 | 44.56 (29.48 + 15.08) | Valence | Arousal | / | / | 0.45 |
| Nogueira et al., 2015 | Not reported | 2 | 91.8 (88.2 + 3.6) | Distressed/anxious/nervous to relaxed/calm/satisfied | / | / | / | Not reported (mentions strongest loadings of min. 0.52) |

Abbreviations: PCA: Principal Component Analysis; PC: Principal Component; KMO: Kaiser–Meyer–Olkin Test for Sampling Adequacy; Bartlett's test: Bartlett’s test of Sphericity.
“/” Information not relevant/applicable, not reported, not possible to extract or unnamed PCs.
1: Method of data suitability as reported in the publication. If other methods than KMO and Bartlett’s test was reported, this was noted (also if not related directly to data suitability).
2: Number of retained PCs, including information on how many PCs were included for analysis or data interpretation, if provided.
3: Percentage of variance of the included PCs explained. Provided in total (percentage for each PC is provided in brackets). If the study included several experiments and/or analyses, these are provided for each part in the cell.
4: The label/name given to the PC as provided in the study. The table only showcase PC1 – PC4, because only one study (Collins et al., 2021) utilised more than four PCs (5).
5: The factor loadings that were reported, or could be extracted, as used for PC interpretation. If specific cut-offs were provided, these are included.
6: Combined PCA (i.e. the QBA data was combined in a PCA also including other parameters such as welfare indicators); values are hence not provided.
* (The study used the same data as Andreasen et al., 2013)

**Table 5.** (*continued*)
